# Effects of donkey milk on UVB-induced skin barrier damage and melanin pigmentation: A network pharmacology and experimental validation study

**DOI:** 10.3389/fnut.2023.1121498

**Published:** 2023-03-09

**Authors:** Anqi Li, Hailun He, Yanjing Chen, Feng Liao, Jie Tang, Li Li, Yumei Fan, Li Li, Lidan Xiong

**Affiliations:** ^1^Cosmetics Safety and Efficacy Evaluation Center, West China Hospital, Sichuan University, Chengdu, Sichuan, China; ^2^NMPA Key Laboratory for Human Evaluation and Big Data of Cosmetics, Chengdu, China; ^3^Department of Dermatology, West China Hospital, Sichuan University, Chengdu, Sichuan, China; ^4^National Engineering Research Center for Gelatin-based Traditional Chinese Medicine, Dong-E-E-Jiao Co. Ltd., Shandong, China; ^5^Laboratory of Pathology, West China Hospital of Sichuan University, Chengdu, Sichuan, China

**Keywords:** donkey milk, UVB, skin barrier function, melanogenesis, network pharmacology

## Abstract

**Introduction:**

Dairy products have long been regarded as a controversial nutrient for the skin. However, a clear demonstration of donkey milk (DM) on skincare is required.

**Methods:**

In this study, spectrum and chemical component analyses were applied to DM. Then, the effects of DM on UVB-induced skin barrier damage and melanin pigmentation were first evaluated *in vitro* and *in vivo*. Cell survival, animal models, and expression of filaggrin (FLG) were determined to confirm the effect of DM on UVB-induced skin barrier damage. Melanogenesis and tyrosinase (TYR) activity were assessed after UVB irradiation to clarify the effect of DM on whitening activities. Further, a network pharmacology method was applied to study the interaction between DM ingredients and UVB-induced skin injury. Meanwhile, an analysis of the melanogenesis molecular target network was developed and validated to predict the melanogenesis regulators in DM.

**Results:**

DM was rich in cholesterols, fatty acids, vitamins and amino acids. The results of evaluation of whitening activities *in vitro* and *in vivo* indicated that DM had a potent inhibitory effect on melanin synthesis. The results of effects of DM on UVB‑induced skin barrier damage indicated that DM inhibited UVB-induced injury and restored skin barrier function *via* up-regulation expression of FLG (filaggrin). The pharmacological network of DM showed that DM regulated steroid biosynthesis and fatty acid metabolism in keratinocytes and 64 melanin targets which the main contributing role of DM might target melanogenesis, cell adhesion molecules (CAMs), and Tumor necrosis factor (TNF) pathway.

**Discussion:**

These results highlight the potential use of DM as a promising agent for whitening and anti-photoaging applications.

## Introduction

Exposure to ultraviolet (UV) radiation from sunlight accounts for a global rise in premature skin aging and skin cancer (1). UV rays are separable into three types through wavelength: UVC is 200–280 nm, UVA is 320–400 nm and 280–320 nm is UVB (2). Since shorter UV wavelengths bring about more terrific impairment to the human body, the impairment induced by UVC is more pernicious than that caused by UVA and UVB (3). UVC has been mostly absorbed by the ozone layer in the atmosphere. Therefore, UVB is the most dominant UV radiation for resulting in wrinkles, laxity, coarseness, and mottled pigmentation (4). In the sunburn response, vasodilation and increased blood flow, endothelial cell activation, formation of “sunburn cells” (i.e., keratinocytes undergoing p53-dependent apoptosis), and release of inflammatory mediators occur in the epidermis and dermis before erythema and edema (5, 6). Proinflammatory cytokines, i.e., TNF-α, PGE2, PGE3, COX-2, IL-6, and IL-8, may play several roles in UVB-induced inflammation, including activation of transcription factors, upregulation of endothelial adhesion molecules, and recruitment of neutrophils to the skin (7–9).

Acute skin damage due to tanning manifests as sunburn (4). Melanogenesis, on the other hand, may protect skin from the damages caused by UV irradiation (10). Exposure to UV radiation, keratinocytes secrete an important melanogenesis regulator, α-melanocyte stimulating hormones (α-MSH), which may trigger the microphthalmia-associated transcription factor (MITF) activation through the melanocortin 1 receptor (MC1R) signaling pathway in melanocytes (11). Then, the tyrosinase (TYR) activity and melanin production are subsequently upregulated in the melanosome. Finally, melanin, which is produced and stored in melanocytes, is transferred to their attached keratinocytes. UVB-mediated pigmentation (delayed tanning) can also be triggered by an inflammatory cascade, suggesting that inflammation and sunburn are also important in the tanning response (12–14). Furthermore, UV damage to the skin triggers inflammation that decreases the expression of genes associated with permeability barrier repair (15). Filaggrin (FLG), which is thought to be a major factor in the skin barrier, is reduced by sunburn (4, 16). During the past decade, safeguarding against UV radiation has been highly studied and was promoted in lots of public health education programs (17). Researchers have frequently concentrated on how to forbid excessive UV exposure, and seldom pay attention to sunburn repairing, post-basking recovery, and pigmentation mechanisms (14, 18).

Milk, one of the most significant provisions for mammals, is the preferred form of feed supplying nutrients and energy (19). Dairy products have been regarded as a conventional nutrient for the skin and milk bath remains popular. Dairy protein allergy, nevertheless, is the most prevailing food allergy in infants, that often experience crossed sensitivity to the present substitute formulae including sheep, goats, milk hydrolysate, and soya bean milk (20). Donkey milk (DM), as a valid natural substitute for cow milk, is similar to human milk in chemical components and organoleptic characteristics (21), which draws our attention. To our knowledge, no allergic reaction to DM has been reported so far. It is said that Cleopatra took DM for a shower to lighten the skin around 3,000 years ago (22). Many milk compositions have shown promise in preclinical studies and have been undergoing active clinical trials (23). DM may benefit overall skin health and cure some skin diseases because DM is rich in vitamin A, vitamin C, niacin, phosphorus, magnesium, zinc, glycine, glutamic acid, ω3-polyunsaturated fatty acids, lipidic prostaglandins, leukotrienes (22), all of which occur in pharmaceuticals and cosmetics.

Until now, the anti-photo damage activities of DM, especially skin barrier protection and melanin production inhibitory activities, have not been reported yet. This paper aimed at exploring the protective effects of DM on UVB-induced skin barrier damage and melanin pigmentation *via in vitro* and *in vivo* studies. In the animal model study, DM was applied topically on the UVB-irradiated dorsum skin of mice. The thickness and integrity of these irradiated skin were evaluated at definite time points. *In vitro* study, we added DM in the culture medium of UVB-irradiated HaCaT cells and observed the viability and protein expression. Meanwhile, B16 cells were applied to evaluate TYR activity and melanogenesis with or without DM after UVB-irradiated. Besides, the mechanisms of DM on UVB-induced skin barrier damage and melanin pigmentation were evaluated *via* a network pharmacology method. Overall, DM has considerable potential as a functional ingredient in food, cosmetic and pharmaceutical applications.

## Materials and methods

### Materials and reagents

DM was provided by Dong-E E-Jiao Co. Ltd (Shandong, China). B16 and HaCaT cell lines were from Kunming Institute of Zoology, Chinese Academy of Sciences. Dulbecco’s modified Eagle’s Medium (DMEM), penicillin and streptomycin solution and trypsin (0.25%) were obtained from HyClone (GE Health Care Life Science, Little Chalfont, Buckinghamshire, United Kingdom). Ascorbic acid (Vitamin C), tyrosinase, and L-dopa were purchased from Sigma (Sigma, United States). Fetal bovine serum (FBS) was from Gibco (Thermo Fisher, Waltham, MA, United States). Phosphate-buffered saline (PBS) was purchased from Zsbio Commerce CO (Zsbio, China). CCK-8 (Cell Counting Kit-8) was gained from Dojindo (Dojindo Laboratories, Kumamoto, Japan). Trizol reagent was acquired from Invitrogen Life Technologies (Carlsbad, CA). FLG primary antibody and corresponding secondary antibody (all from rabbit, 1: 2000) were procured from Abcam company (Abcam, United Kingdom). HE Stain assay kit was procured from Solarbio (Solarbio Inc., China). All other chemicals and solvents were of analytical grade.

Microplate spectrophotometer was Bio-Rad (Bio-Rad Inc., Hercules, CA, United States). Chromatography instruments were Agilent 7890A, Agilent ICP-OES5110, Agilent 1,200, and Thermo U3000, respectively. High-speed amino acid analyzer is Hitachi L-8900. Fluoro spectrophotometries are AFC062 and AFC045. Potentiometric titrator is AFC057.

### Determination of main active ingredients in donkey milk

#### Total amino acids composition analysis

AAs concentrations in DM were analyzed by a Hitachi L-8900 AAs analyzer (Hitachi, Ltd., Japan). A mixture of basic, acid, and neutral AAs of known concentrations (Sigma Chemical Co., St. Louis, MO) was used as standard. The samples were hydrolyzed in 6 mol HCl at 110°C for 22 h and filtered through a filter (pore size, 0.22 μM). AAs were derived through reactions with the ninhydrin reagent and detected by the absorbances at 440 nm (proline and hydroxyproline) or 570 nm (total AAs except for proline and hydroxyproline).

#### Determination of fatty acids content

Fatty acids of DM were determined by Gas chromatography (GC) analysis. The fatty acids were obtained with sodium hydroxide in methanol and injected into an Agilent 7890A gas-chromatograph device (Agilent Technologies, United States), equipped with a flame ionization detector. The chromatographic column (Supelco SP-2560, China) was performed. Purified helium was used as a carrier gas with a split ratio of 1: 100. A 1.0 μl aliquot of each sample was injected at an initial temperature of 100°C and held constant for 13 min before being increased to 180°C at 10°C/min and held for 6 min, then ramped to 200°C at 1°C/min held for 20 min and then increased to 230°C at 4°C/min held for 10.5 min. The injector and detector temperatures were set at 270°C and 280°C, respectively.

#### Determination of mineral content

Determination of Ca, Fe, K, Mg, Na, and Zn was carried out with an inductively coupled plasma optical emission spectrometer (Agilent Technologies, United States). For plasma generation, nebulization, and auxiliary gas, argon with a purity of 99.996% was used. Digestion of samples was also performed using HNO_3_ (5% v/v) in a microwave dissolver (MARS, USA). After the digestion procedure, clear solutions were obtained, and the analytes were determined by ICP-OES. The ICP-OES operating conditions are listed in Supplementary Table S1.

#### Determination of cholesterol content

The cholesterol content was determined according to the National Standards of the PRC. DM was saponified with methanolic potassium hydroxide, and the unsaponifiable matter was extracted by ligarine and diethyl ether, separated, and determined by HPLC. Analysis of cholesterol was performed by Agilent 1,200 (Agilent Technologies, United States) adapted a ZORBAX SB-C18 (4.6 mm × 150 mm, 5.0 μM) column and detected at 205 nm. The samples were filtered before injection (Millipore 0.45 μM). Injected volume was 50 μl, flow rate 1.0 ml/min, isocratic mode with methanol.

The determination of other chemical components (vitamin C, vitamin D2, vitamin D3, taurine, phosphorus, and chloride) of DM powder was provided in the section Supplementary Materials and Methods.

### Cell viability assay

Cytotoxic effect of DM on B16 or HaCaT cells was determined by the CCK-8 assay. The B16 or HaCaT cells were seeded at 2–8 × 10^4^ cells/well in 24 well plates and incubated in a humidified incubator at 37°C under 5% CO_2_ for 24 h. The cells then were cultured for 24 h with or without DM (0.1–25 mg/mL). Cell survival was calculated as the percentages of that of control. Each sample was tested for three independent analyses.

### Evaluation of donkey milk on melanin pigmentation *in vitro*

#### Determination of melanin content

B16 cells were seeded at a density of 2–5 × 10^5^ cells/mL in 6-well plates and incubated for 24 h. Cells were then exposed to increasing doses of DM or ascorbic acid (VC) for 48 h in the presence or absence of 100 nM α-MSH. Then harvesting and centrifugation for 10 min at 4°C, the cells were dissolved in 1 M NaOH at 80°C for 1 h. The melanin content was gauged by the absorbance of microplate reader at 405 nm.

#### Assay of tyrosinase activity

The cells were seeded at a density of 2–5 × 10^5^ cells/mL in 6-well plates and cultured for 24 h. Cells were then exposed to increasing doses of DM or VC for 48 h in the presence or absence of 100 nM α-MSH, Then, cells were washed twice with PBS and lysed in 1.0% Triton X in a refrigerator at-80°C for 30 min. 0.5% L-DOPA was added to each cell lysate and incubated at 37°C for 3 h. All the values of absorbance were gauged with a spectrophotometer at 475 nm. The inhibitory activity of TYR activity was expressed as inhibition ratios of that of control.

### Tyrosinase, dopachrome tautomerase, tyrosinase-related protein 1, and microphthalmia-associated transcription factor mRNA expression assay

An amount of 1 × 10^5^ B16 cells per well were cultured in 24-well plates for 24 h and then incubated with PBS after washing for twice. Cells were then exposed to increasing doses of DM for 48 h in the presence or absence of 100 nM α-MSH. The Trizol method was applied to extract total cellular RNA according to the manufacturer’s instruction. NovoScript Kit (Novoproptein, China) was then used for the amplification with real-time PCR (Bio-Rad Inc., United States). The 2-ΔCT approach was performed to investigate gene Tyr, Dopachrome tautomerase (Dct), Tyrosinase-related protein 1 (TYRP1), MITF expression and β-actin mRNA served as an endogenous control to evaluate the relative expression levels of target mRNAs. The sequences of primers were listed in Table 1.

**Table 1:** The sequences of primers used for reverse transcription.

Gene	Nucleotide sequence	Size
TYR	F:5′-CCTTCTGTCCAGTGCACCAT-3′	20	R:5′- TCCGCAGTTGAAACCCATGA-3′	20
DCT	F: 5′-CTTGGGGTTGCTGGCTTTTC-3′	20	R:5′- CGCTGAAGAGTTCCACCTGT-3′	20
TYRP1	F:5′- GCTTCACTTGCTGGAACACA-3′	20	R:5′-CGCAGGCCTCTAAGATACGA-3′	20
MITF	F:5′-TGCACTGGGGAGAAGTTGAT-3′	20	R:5′-GCTGCGGACCATACAGAAAG-3′	20
β-actin	F:5′-ACAGCTGAGAGGGAAATCGTG-3′	21	R:5′-AGAGGTCTTTACGGATGTCAACG-3′	23

F, forward primer; R, reverse primer.

### Evaluation of donkey milk on UVB-induced skin barrier damage

#### Filaggrin protein expression assay

An amount of 5 × 10^5^ HaCaT cells per well were cultured in 6-well plates for 24 h. After PBS washing three times, the cells were covered with PBS and a dose of 20 mJ/cm^2^ UVB irradiation. The cells were then cultivated in serum-free DMEM culture medium with DM added at varying concentrations for 24 h, or untreated (control， with neither UVB radiation nor DM supplement). The HaCaT cells were then acquired on an ice plate and added the lysis buffer to lyse for 30 min. Cellular extracts were then centrifuged at temperature 4°C for 15. The BCA assay (Beyotime, China) was then applied to evaluate collected total proteins’ amount. Commercial SDS-PAGE gels (Beyotime, China) were utilized to separate whole proteins and protein bands, the proteins were then electro-transferred to PVDF membranes (Millipore, United States). After transferring, PVDF membranes were blocked for 30 min with the quick confining liquid (Beyotime, China). To probe corresponding target proteins, PVDF membranes were incubated with GAPDH and filaggrin (FLG) antibodies for 24 h at 4°C, subsequently incubated with secondary antibody for 1 h at 20°C. The iBright system (iBright FL1500, Thermo Fisher, USA) was employed to assess the protein bands in this study. Relative expression of objective protein (GAPDH as an internal control) was observed by electrophoresis bands’ optical density and calculated with Image J (National Institutes of Health, Germany).

#### *In vivo* experiments

Six-week-old female C57BL/6 mice (Chengdu Dashuo Inc., China) were raised under standard animal husbandry conditions. This study was approved by the Ethical Committee of the West China Hospital of Sichuan University (Chengdu, China). The mice were separated randomly into five groups: a negative control group (n = 3), which was exposed to no UVB irradiation; a positive control group (n = 3), which received UVB irradiation and with no treatments; a hydrocortisone group (n = 3), which received UVB rays and treated with hydrocortisone cream; a concentration of 5 mg/ml DM treatment group (n = 3), which was exposed to UVB irradiation and treated with 5 mg/ml DM; a concentration of 10 mg/ml DM treatment group (n = 3), which was exposed to UVB irradiation and treated with 10 mg/ml DM. those external productions were applied once a day. After the treatment process, the animals were sacrificed on the 7th day, and skin lesion specimens were immersed in 4% paraformaldehyde. Hematoxylin and eosin (H&E) staining was used to demonstrate the general histopathological variations in the skin.

### Network pharmacology study

#### Relevant targets data collection

The test report of DM ingredients was released as described above. GeneCards^1^ (updated on December, 2019) (24), DrugBank^2^ (updated on December, 2019) (25), and ChEMBL^3^ (updated on Dec, 2019) were used. In order to acquire integrated and accurate data, substantial work of data mining and literature searching needed to be explored to ascertain the construction of the database.

#### Network analysis

The targets databases for ingredients of DM and UVB-induced skin barrier damage and melanin pigmentation were utilized to mine the potential UVB-protective targets. The ingredients of DM and relevant targets databases were utilized to mine the targets. To determine the connection between ingredients and target for DM, a network study was developed using STRING^4^ (updated on August, 2019) and plotted using Cytoscape^5^ (version 3.7.1) (26). We used Cytoscape 3.7.1 software to construct protein–protein (PPI) and component-target interaction networks. Target protein molecules were displayed by “nodes” and interrelationships by “edges.” With excellent visual interface, the interaction between components and targets can be clearly shown. Pasted targets into the list of genes on the right and submitted them. We performed several gene ontology (GO) analyses (*p* < 0.05) and used Kyoto Encyclopedia of Genes and Genomes (KEGG) automatic annotation database^6^ (KAAS) to analyze the obtained targets and related signaling pathways (p < 0.05).

#### Statistical analysis

The results were shown as the mean ± standard error (mean ± S). Data were determined by one-way analysis of variance (one-way ANOVA) and Kruskal-Wallis H rank sum test. A *value of p* less than 0.01 was considered statistically significant. IBM SPSS Statistics 23 was applied.

## Results

### Chemical composition analysis of main active ingredients in donkey milk

**Figure 1:**
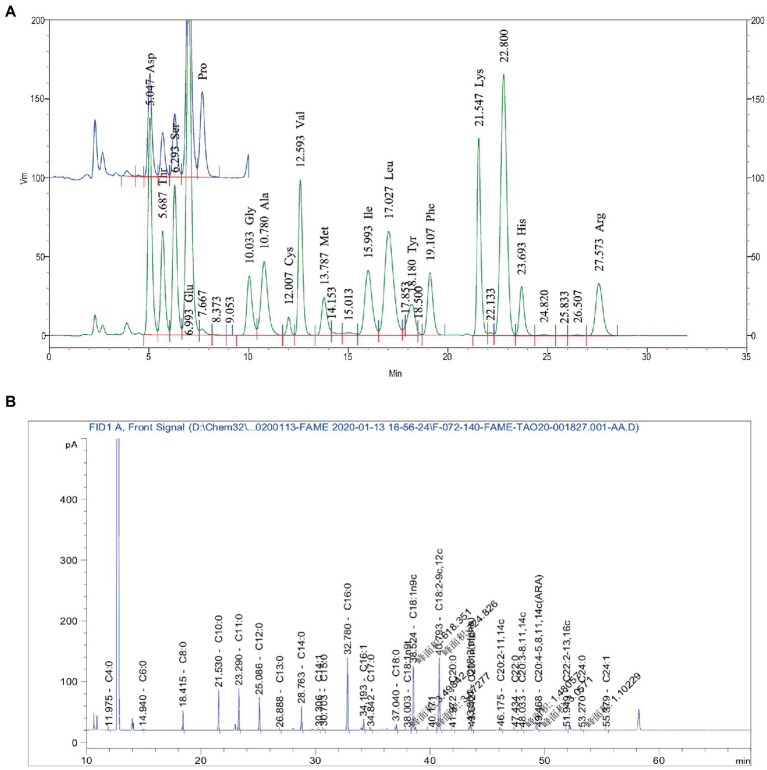
Main Active Ingredients in DM. **(A)** Main amino acids. **(B)** Main fatty acid.

Table 2 presents the experimental data on the main active components content. As shown in Table 2, the content of mineral was the highest, followed by the amino acids and fatty acids. Of the 16 amino acids identified, the most abundant were glutamic acid (Glu), Aspartic acid (Asp), leucine (Leu), lysine (Lys), valine (Val), and Arginine (Arg), which accounted for over 66% of the total amino acids (Figure 1A). The results showed that 28 kinds of fatty acids were detected in DM (Figure 1B). The content of fatty acids in DM powder was 4.5%. The content of saturated fatty acids and unsaturated fatty acids was 2.16 and 2.34%, respectively (1.12% for unsaturated fatty acids and 1.22% for polyunsaturated fatty acids). The content of unsaturated fatty acids of DM, including myristoleic (C14:1), palmitoleic (C16:1), trans-elaidic (C18:1n9t), oleic (C18:1n9c), linoleic (C18:2-9c,12c), α-linolenic acid (C18:3; ALA), cis 11-eicosenoic acid (C20:1n11c), all cis-11,14-eicosadienoic acid (C20:2–11,14c), all cis-8,11,14-eicosatrienoic acid (C20:3–8,11,14c), all cis-5,8,11,14-eicosateraenoic acid (C20:4–5,8,11,14c; ARA), all cis-13,16-docosadienoic acid (C22:2–13,16c), nervonic (C24:1).

**Table 2:** The main contents of DM.

Items	Unit	Testing methods	Molecular formula	Molecular weight	Values
MUFA	g/100 g	GC	–	–	1.6
PUFA	g/100 g	GC	–	–	0.25
SFA	g/100 g	GC	–	–	3.08
LA	g/100 g	GC	C_18_H_30_O_2_	278.43	0.21
ALA	g/100 g	GC	C_18_H_30_O_2_	278.43	0.08
linoleic acid	g/100 g	GC	C_18_H_32_O_2_	280.44	1.12
AA	g/100 g	GC	C_20_H_32_O_2_	304.46	ND
DHA	g/100 g	GC	C_22_H_30_O_2_	328.48	ND
Vitamin C	mg/100 g	Fluoro spectrophotometry	C_6_H_8_O_6_	176.12	48.1
Vitamin D_2_	ug/100 g	HPLC	C_28_H_44_O	396.65	ND
Vitamin D_3_	ug/100 g	HPLC	C_27_H_44_O	384.64	ND
P	mg/100 g	Fluoro spectrophotometry	–	–	580
Cl	g/100 g	Zeta	–	–	0.58
Ca	mg/kg	ICP-OES	–	–	8.81*10^3^
K	mg/kg	ICP-OES	–	–	8.27*10^3^
Na	mg/kg	ICP-OES	–	–	2.59*10^3^
Mg	mg/kg	ICP-OES	–	–	826
Fe	mg/kg	ICP-OES	–	–	ND
Zn	mg/kg	ICP-OES	–	–	22.7
Cho	mg/100 g	HPLC	C_27_H_46_O	386.65	21.7
Tau	mg/100 g	HPLC	C_2_H_7_NO_3_S	125.15	12.3
Glu	g/100 g	HPLC	C_5_H_9_NO_4_	147.13	3.48
Gly	g/100 g	HPLC	C_2_H_5_NO_2_	75.07	0.33
Ile	g/100 g	HPLC	C_6_H_13_NO_2_	131.17	0.86
Tyr	g/100 g	HPLC	C_9_H_11_NO_3_	181.19	0.55
Lys	g/100 g	HPLC	C_6_H_14_N_2_O_2_	146.19	1.33
Arg	g/100 g	HPLC	C_6_H_14_N_4_O_2_	174.20	0.9
Thr	g/100 g	HPLC	C_4_H_9_NO_3_	119.12	0.72
Ser	g/100 g	HPLC	C_3_H_7_NO_3_	105.09	0.91
Pro	g/100 g	HPLC	C_5_H_9_NO_2_	115.13	1.41
Ala	g/100 g	HPLC	C_3_H_7_NO_2_	89.09	0.61
Met	g/100 g	HPLC	C_5_H_11_O_2_NS	149.21	0.41
Leu	g/100 g	HPLC	C_6_H_13_NO_2_	131.18	1.62
Phe	g/100 g	HPLC	C_9_H_11_NO_2_	165.19	0.81
Val	g/100 g	HPLC	C_5_H_11_NO_2_	117.15	1.06
Asp	g/100 g	HPLC	C_4_H_7_NO_4_	133.10	1.73
His	g/100 g	HPLC	C_6_H_9_N_3_O_2_	155.00	0.49

MUFA, monounsaturated fatty acid; PUFA, polyunsaturated fatty acid; SFA, saturated fatty acid; LA, linolenic acid; ALA, α-linolenic acid; DHA, docosahexaenoic acid; AA, arochidonic acid oil; Cho, cholesterol; Tau, taurine; Glu, glutamic acid; Gly, glycine; Cys, cystine; IIe, isoleucine; Tyr, tyrosine; Lys, lysine; Arg, arginine; Trp, tryptophan; Thr, threonine; Ser, serine; Pro, proline; Ala, alanine; Met, methionine; Leu, leucine; Phe, phenylacetic acid; Val, valine; Asp, aspartic acid; His, histidine; ICP-OES, inductively coupled plasma optical emission spectrometry; HPLC, high performance liquid chromatography.

The determination of other chemical components of DM was provided in the section Supplementary Results.

### Evaluation of whitening activities of donkey milk *in vitro*

#### The cytotoxicity of donkey milk in B16 cells

**Figure 2:**
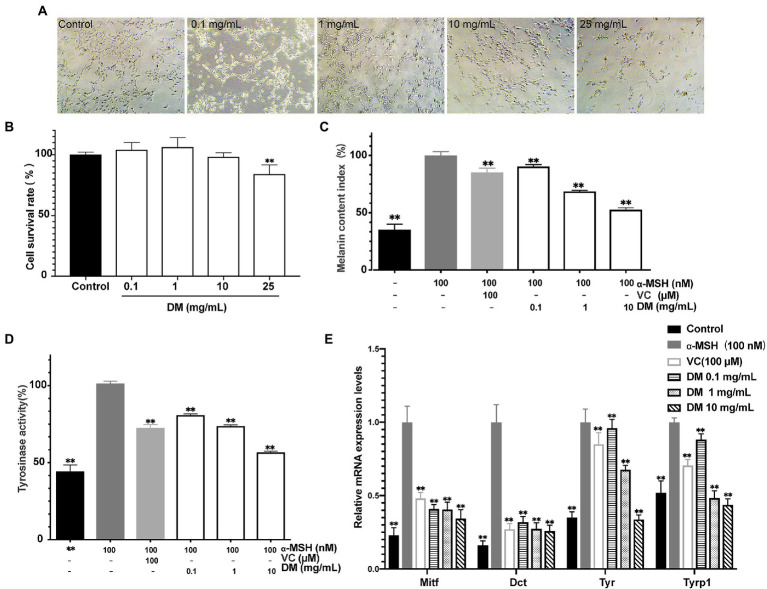
Whitening efficacy *in vitro*. **(A)** Light microscope image. **(B)** Effects of DM on B16 cell viability. **(C)** Effects of DM on melanin synthesis in B16 cells. **(D)** Effects of DM on TYR activity in B16 cells. **(E)** Effects of DM on expression of Mitf, Dct, Tyr, and Tyrp1 in B16 cells. The asterisk (**) indicated a significant difference (*p* < 0.01) compared to the control of **(B)**. The asterisk (**) indicated a significant difference (*p* < 0.01) compared to the 100 nM ɑ-MSH of **(C–E)**.

The cytotoxic effects of DM were evaluated by CCK-8 assay and light microscopic observation (Figures 2A,B). At DM concentrations of 25 mg/ml, light microscopy revealed significant toxicity as cells became round shaped and uniformly detached from the surface. CCK-8 assay demonstrated that the difference between 25 mg/ml DM and control group was statistically significant (*P*<0.01). Overall, a safe concentration of DM of under 10 mg/ml was used for the next stage of the experiment.

#### Effect of donkey milk on melanin content in B16 cells

To explore the effect of DM, an experiment *in vitro* was applied to check whether DM activated or inhibited melanogenesis in cells that turn related-genes and proteins on and off. Treatment with various dosages of DM (0.1, 1, 10 mg/ml) showed inhibitory effect on melanogenesis in a dose-dependent manner in the B16 cells. The melanin content was 90.36% at 0.1 mg/ml, 68.67% at 1 mg/ml, and 52.81% at 10 mg/ml compared with the level of the 100 nM α-MSH group (Figure 2C). Meanwhile, B16 cells were treated with 1 mM VC as a positive standard (Figure 2C). The results indicated that DM had a potent inhibitory effect on melanin synthesis in B16 cells.

#### Effect of donkey milk on tyrosinase activity in B16 cells

As presented in Figure 2D, DM inhibited the TYR activity notably in a dose-dependent manner, by 81.78, 72.12, and 56.57% compared with 100 nM α-MSH at concentrations of 0.1, 1, and 10 mg/ml, respectively. Meanwhile, the TYR activity was decreased to 71.02% after the cells were exposed to 1 mM VC.

#### Effect of donkey milk on the expression of melanogenesis-related genes in B16 cells

As shown in Figure 2E, inhibition of Tyr, Trp1, Dct (Trp2), and Mitf expression with 1 mM VC was observed by 0.95-, 0.88-, 0.17-and 0.48-fold of 100 nM α-MSH, respectively. The inhibitory effects of DM (0.1,1, 1 mg/ml) on mRNA expression of Mitf were equivalent to the effects of 1 mM VC, which served as a well-known effective melanogenesis inhibitor, while the effects of 10 mg/ml DM was superior to that of 1 mM VC (*P*<0.01). DM (0.1, 1, 1 mg/mL) inhibited mRNA expression of Dct in a dose-dependent manner. In addition, the inhibitory effects of 0.1 mg/ml DM on TYR and TRP1 were inferior to the effects of 1 mM VC, while DM (1 and 10 mg/ml) on TYR and TRP1 was superior to the effects of 1 mM VC (*P*<0.01), further indicating that DM has a potential whitening effect.

### Effects of donkey milk on UVB-induced skin barrier damage

#### The protective effect on UVB-induced damage of donkey milk in mouse

**Figure 3:**
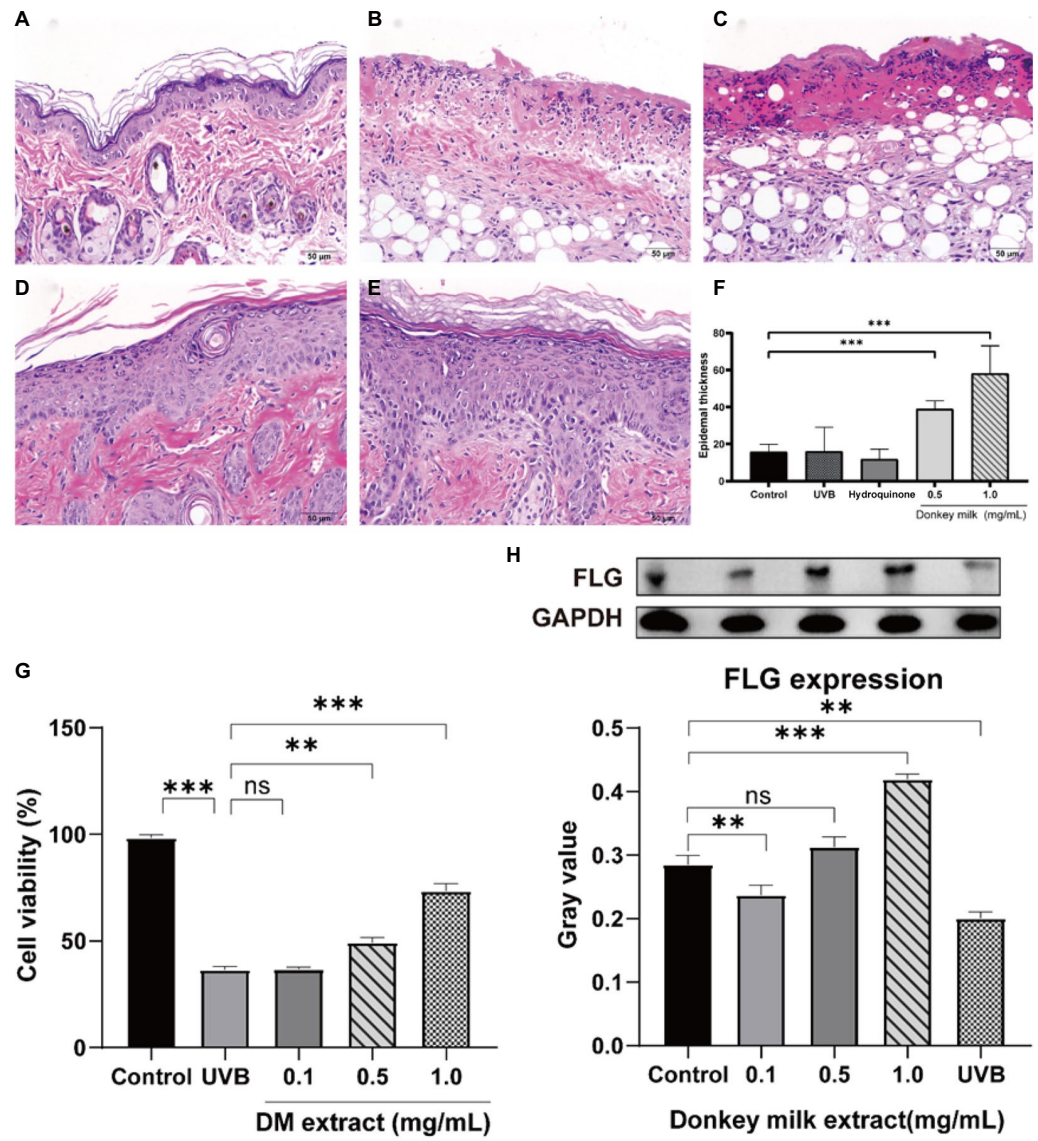
Effects of DM on UVB-induced skin barrier damage. The protective effect of DM in mouse through HE staining. **(A)** Control group. **(B)** UVB group. **(C)** Hydroquinone group. **(D)** 0.5 mg/mL DM group. **(E)** 1 mg/mL DM group. **(F)** Epidermal thickness. **(G)** Effects of DM on HaCaT cell viability after UVB exposure. **(H)** Effects of DM on expression of FLG in HaCaT cells. The asterisk (** and ***) indicated a significant difference (*p* < 0.01 and *p* < 0.001), the symbol (ns) indicated no significant difference.

After UVB irradiation, mice were topically treated with DM for 7 days. Figures 3A–F showed histological results of mouse skin exposed to UV and then treated with DM. After staining by hematoxylin and eosin (H&E), the control group demonstrated normal cutaneous histology which had the integrated epidermal structure and basement membrane zone without inflammatory cell infiltration. When exposed to UVB only, there was a significant acanthosis with liquefaction degeneration in basal cells, consistent with the exfoliation of epidermal and stratum corneum, severe inflammatory cells infiltration could be observed both in derma and epidermis. Hyperplasia of the spinosum and strata granulosum was illustrated as the histological alterations in UVB-irradiated skin. Compared to control group, there were significant differences when treated with DM from H&E photomicrographs. Considerable hyperplastic epidermis alterations were observed by after UVB radiation, and an increased thickness of epidermis in DM groups were observed, suggesting that DM provided a protective effect after UVB exposure *via* enhancing the structure of keratinocytes and the epidermal thickness. Compared to the group of hydrocortisone, the DM group showed less irritation.

#### Effect of donkey milk on cell viability after UVB irradiation in HaCaT cells

After UVB (20 mJ/cm^2^) exposure, cell survival rate of HaCaT cells gradually declined to 36.2% after 24 h (Figure 3G). A concentration-dependent protective effect was indicated *via* analysis of cells survival while treated with DM. In comparison with control group (without UVB exposure), the amount of HaCaT cells after exposed to UVB was declined to exactly 73.1, 49.6 and 35.7%, after treated to DM for 1, 0.5 and 0.1 mg/ml, respectively. And the cell viability of UVB-exposed group without DM was only 36.2%. Compared to UVB group, the DM (0.1 mg/ml) group indicated no significant difference (*p* > 0.05). The results for viability of HaCaT cells after UVB exposure indicated that DM could play an important role in protecting keratinocytes against UVB injury.

#### Effect of donkey milk on the expression of filaggrin after UVB irradiation in HaCaT cells

After exposure to UVB (20 mJ/cm^2^), HaCaT cells were incubated with 0, 0.1, 0.5 and 1 mg/ml DM for 24 h, then cells were collected. Filaggrin (FLG) expression, one of the key structural components of the epidermal barrier, was detected through Western blot. It illustrated that UVB irradiation led to down-regulation in FLG expression compared to untreated cells (*p* < 0.05, Figure 3H). Usage of DM (0.5 and 1 mg/ml) up-regulated the FLG expression vs. UVB group (*p* < 0.05). These results indicated DM inhibited UVB-induced injury and restored skin barrier function *via* up-regulating the expression of FLG.

### Network pharmacology analysis

#### Donkey milk—target (ingredient) -melanin related targets network

**Figure 4:**
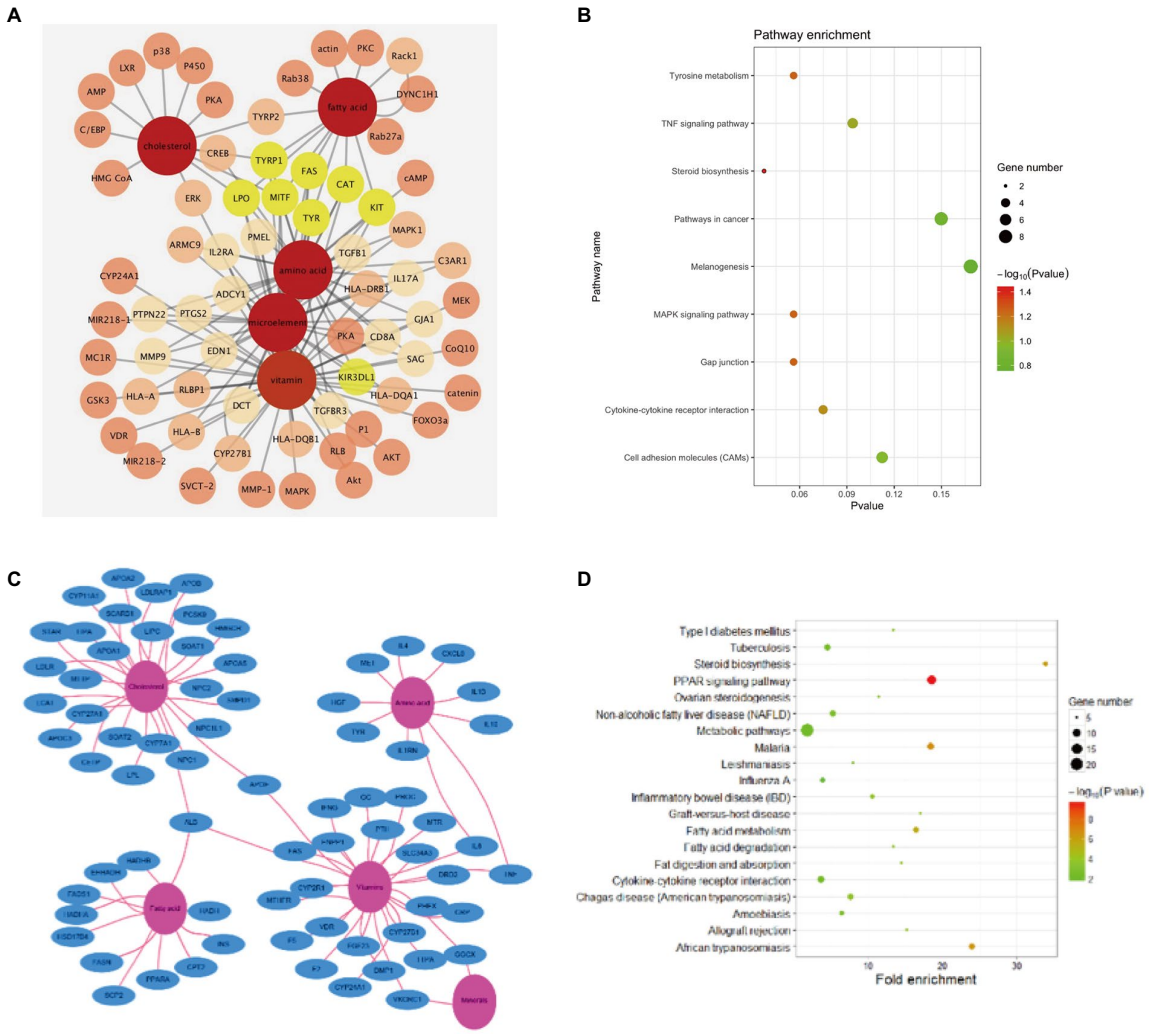
Network pharmacology analysis. **(A)** Components-melanin related targets interaction network. **(B)** Top 9 KEGG enrichment in DM components-melanin network pharmacology. **(C)** Components-skin barrier related targets interaction network. **(D)** Top 20 KEGG enrichment in DM components-skin barrier network pharmacology.

To explore the relationship between DM and the effect of lightening pigmentations, a DM-target (ingredient)—melanin-related targets pharmacological network was used. A molecular target network was developed and validated to predict the melanogenesis regulators related to 64 melanin targets. Among the constructed and visualized target prediction database, 64 DM-related targets were observed, including TYR, TRP1, DCT (TRP2), and MITF which confirmed a high correlation with melanogenesis. There were several dozens of other proteins seemingly unrelated such as fatty acid synthetase (FAS), catalase (CAT), KIT Proto-Oncogene, Receptor Tyrosine Kinase (KIT), and Lactoperoxidase (LPO). However, using Cytoscape to construct PPI network and component-target interaction network, we found that 53 PPI-related targets were mapped after 11 protein factors were excluded (Figure 4A). According to PPI enrichment *p* < 1.0e-16, three of the most interacting nodes were TYR, TRP1, DCT (TRP2), while MITF, FAS, CAT, KIT, LPO and killer cell immunoglobulin (Ig) like receptor, KIR3DL1 were the most potent factors. In accordance with the effect, the targets ranged from strong to weak. Component-target interaction network showed that 2 major categories of related active ingredients interacted with 53 gene targets. KEGG analysis was applied to enrich the related signaling pathways (*p* < 0.05), melanogenesis, cell adhesion molecules (CAMs), TNF signaling pathway, pathways in cancer tyrosine metabolism, gap junction, MAPK pathway, steroid biosynthesis, and cytokine-cytokine receptor interaction were dominant (Figure 4B).

To investigate the protective mechanism of DM on skin recovery after UVB exposure, a DM-target (ingredient)-UVB related targets pharmacological network was used. The potential targets data of DM were obtained from GeneCards. The potential targets data of UVB injury were obtained from mRNA sequencing of HaCaT cells after 20 mJ/cm^2^ UVB exposure. There were 72 potential targets (score > 50) related to the protective effect of DM. The top 10 of them were APOA1, HADHA, HGF, LDLR, NPC1, F2, APOA2, APOB, IL10, APOE, which were related to lipid metabolism and inflammatory reaction. Ingredient-target gene interactions’ network diagrams were plotted to employ Cytoscape (Figure 4C). As shown above, DM was rich in cholesterols, fatty acids, vitamins, minerals and amino acids. Component-target interaction network showed that 4 major categories of related active ingredients interacted with 72 gene targets, which were cholesterols, fatty acids, amino acids, and vitamins, respectively (Figure 4C). KEGG analysis was utilized to enrich the relevant signal pathways (*p* < 0.05), and the signal pathway analysis indicated that the protective effects of DM compound against UVB-induced changes might aim at the metabolic pathway, PPAR pathway, fatty acid metabolism, and steroid biosynthesis (Figure 4D). These signal pathways were associated with cell proliferation and metabolism.

## Discussion

Exposure to UVB has psychological and physical benefits, especially in the synthesis of vitamin D_3_ and the prevention of diseases like osteoporosis (27). However, UVB is responsible for photocarcinogenesis and sunburn response (28). Recent investigations have disclosed UVB-induced skin injury’s pathology, like sunburn, photoaging, and skin cancer utilizing cells, animals, and human studies (4, 29, 30). Previous studies also have shown that UVB affected epidermal morphology, disrupted the skin barrier, increased transepidermal water loss, and decreased stratum corneum hydration (31). Initially, melanin pigmentation plays a dual role in skin:it is suggested to render photoprotection from the DNA-damaging effects of UV (32, 33) while leading to acquired hyperpigmentation disorders such as melasma (34, 35). In this context, consumers have an increasing requirement for quality natural cosmetic material. In this study, we found that DM protected against sunburn and tanning. Milk has been used to treat skin wounds for thousands of years (36, 37). In the past few years, researchers’ attention has been attracted to milk products due to several bioactive components’ plenteous presence (21). In this study, HPLC was employed to evaluate the composition of DM. A network pharmacology method was used to find out the potential mechanism and active compounds in DM for restoring skin barrier and pigmentation UVB-induced detriment.

By network prediction, we could find that DM was closely related to melanin metabolism. HPLC analysis revealed that DM is rich in cholesterol, fatty acids, vitamins and amino acids.DM is rich in leucine, lysine, glutamic, isoleucine, threonine, tyrosine, serine, and valine. It has been reported that alanine, glycine, phenylalanine, and aspartic acid were shown to have different effects against melanin contents and TYR activity in B16 melanoma cells according to their chemical structures or their combinations (38). Another study showed glycine hydroxamate downregulated melanin synthesis and TYR activity through activating cAMP/ PKA pathways (39). As a tripeptide component, glutathione serves long as an intravenous anti-pigmentation product by inhibiting TYR activity (40). Moreover, nicotinic acid hydroxamate inhibited the TYR activity and melanogenesis by downregulating the MEK/ ERK and AKT/ GSK3β pathways (41). Besides, the effect of cholesterol, fatty acids, and microelement in DM on lightening pigmentation has been controversial. Some reports demonstrated that fatty acids are able to regulate the post-Golgi proteasomal degradation in ubiquitinated TYR (42). Briefly, linoleic acid and docosahexaenoic acid (DHA) decrease melanin levels, while palmitic acid (PA) increases melanin levels (43). In addition, trace elements such as calcium (Ca), magnesium (Mg), copper (Cu) and zinc (Zn) are a kind of important nutrients, which participate in the body’s metabolism as the components or activators of enzymes and receptors (44). Additionally, TYR with copper binding is the rate-limiting enzyme in melanin biosynthesis and first catalyzes hydroxylation (45). In the present study, a molecular target network was developed and validated to predict the melanogenesis regulators related to 64 melanin targets. DM was evaluated for TYR activity and melanogenesis *in vitro* with experimental validation. Thus, DM was considered to have the potential skin-whitening effect and may be supposed to develop as a safe potentially depigmented agent.

By network prediction, we could find that the nutritious ingredients of DM are the indispensable base of skin barrier reconstruction and keratinocytes survival from UVB exposure. The lipid fraction of DM consists of several nutritional significant components, such as phospholipids and polyunsaturated fatty acids. DM lipids’ importance in skin structure and skin barrier function has been revealed by continued research. Researchers fed hairless mice with milk phospholipids, and discovered mice supplemented with more phospholipids showed higher concentrations of covalently-bound ω-hydroxy ceramides and an improved skin barrier function due to skin inflammation’s suppression (19). Our results revealed that the efficiency of DM is dependent on the concentration, and the potential target genes are APOA1, HADHA, HGF, LDLR, NPC1, F2, APOA2, APOB, IL10, and APOE. Apolipoprotein A-II is the second most plenteous protein in high-density lipoprotein particles, and it is related to lipid metabolism (45). HADHA is related to mitochondrial function and phospholipid metabolism and HADHA’s loss leads to long-chain fatty acid accumulation (46). In summary, APOA1, APOA2, APOB, APOE, HADHA, and LDLR are genes related to lipid metabolism, which is important for keratinocytes proliferation and stratum corneum lipids formation. The significant beneficial effect of DM on sunburn can be explained. Furthermore, we observed that DM can reduce UVB-induced injury by increasing HaCaT cells’ survival. In addition, DM can restore the skin barrier function by increasing the expression of FLG in keratinocytes and epidermal thickness of C57BL/6 mouse skin after UVB exposure. Changes in epidermal structural proteins like FLG are frequently related to damage to cutaneous barrier function. FLG play a fundamental part in skin barrier function, and gene mutations of FLG are usually associated with the deterioration of atopic dermatitis and ichthyosis vulgaris (47, 48).Excessive exposure to sunlight can diminish epidermal FLG and result in an acquired filaggrin insufficiency (49), which is consistent with our results.

## Conclusion

In this study, we suggested that DM help the skin restore after UVB exposure. We conducted *in vitro* tests to reveal that DM was protected against sunburn and tanning. The whitening effect was mainly reflected in the good inhibitory effect of DM on synthesis of melanin, tyrosinase activity, and related gene expression. DM could not only prevent UVB-induced adverse effects but also restore skin barrier function by increasing FLG’s expression and regulating metabolism procedures such as lipid and steroid metabolism. Hence, DM is desirable for skin care cosmetics against UVB-induced skin barrier damage and melanin pigmentation.

## Data availability statement

The original contributions presented in the study are included in the article/Supplementary material, further inquiries can be directed to the corresponding authors.

## Ethics statement

The animal study was reviewed and approved by Animal Ethical and Welfare, West China Hospital, Sichuan University.

## Author contributions

AL, HH, and YC: data curation. JT and YF: formal analysis. FL: funding acquisition. LL (6th author) and LX: project administration, supervision, writing—review and editing, and conceptualization. AL, YC, and LX: investigation, validation, writing—original draft, and methodology. All authors have read and agreed to the published version of the manuscript.

## Funding

We appreciatively acknowledge the financial support offered by China National Natural Science Foundation (No. 81673084) and 1.3.5 project for disciplines of excellence, West China Hospital, Sichuan University.

## Conflict of interest

FL and YF were employed by Dong-E-E-Jiao Co. Ltd.

The remaining authors declare that the research was conducted in the absence of any commercial or financial relationships that could be construed as a potential conflict of interest.

The reviewer RD declared a shared affiliation with the authors LX, AL, HH, YC, JT, and LL to the handling editor at the time of review.

## Publisher’s note

All claims expressed in this article are solely those of the authors and do not necessarily represent those of their affiliated organizations, or those of the publisher, the editors and the reviewers. Any product that may be evaluated in this article, or claim that may be made by its manufacturer, is not guaranteed or endorsed by the publisher.

## Supplementary material

The Supplementary material for this article can be found online at: https://www.frontiersin.org/articles/10.3389/fnut.2023.1121498/full#supplementary-material

## Footnotes

^1^ https://www.genecards.org/

^2^ https://www.drugbank.ca/

^3^ https://www.ebi.ac.uk/chembl/

^4^ https://string-db.org/

^5^ https://cytoscape.org/

^6^ https://www.kegg.jp/blastkoala/

## References

[1] Wei J Shi Q Xiong L Xin G Yi T Xiao Y. Transcriptome profiling of cells exposed to particular and intense electromagnetic radiation emitted by the “SG-III” prototype laser facility. Sci Rep. (2021) 11:1–14. doi: 10.1038/s41598-021-81642-5, PMID: 33479397 PMC7820428

[2] Mahmoud BH Ruvolo E Hexsel CL Liu Y Owen MR Kollias N. Impact of long-wavelength UVA and visible light on melano competent skin. J Invest Dermatol. (2010) 130:2092–7. doi: 10.1038/jid.2010.95, PMID: 20410914

[3] Fu Y Wan R Yang L Xiong L Hu J Tang J. Propolis inspired sunscreens for efficient UV-protection and skin barrier maintenance. Nano Res. (2022) 15:8237–46. doi: 10.1007/s12274-022-4434-z

[4] Matsui M Tanaka K Higashiguchi N Okawa H Yamada Y Tanaka K. Protective and therapeutic effects of fucoxanthin against sunburn caused by UV irradiation. J Pharmacol Sci. (2016) 132:55–64. doi: 10.1016/j.jphs.2016.08.004, PMID: 27590588

[5] Nicolaou A Masoodi M Gledhill K Haylett AK Thody AJ Tobin DJ. The eicosanoid response to high dose UVR exposure of individuals prone and resistant to sunburn. Photochem Photobiol Sci. (2012) 11:371–80. doi: 10.1039/c1pp05272a, PMID: 22173231

[6] Nicolaou A Pilkington SM Rhodes LE. Ultraviolet-radiation induced skin inflammation: dissecting the role of bioactive lipids. Chem Phys Lipids. (2011) 164:535–43. doi: 10.1016/j.chemphyslip.2011.04.005, PMID: 21524643

[7] Vogeley C Esser C Tüting T Krutmann J Haarmann-Stemmann T. Role of the aryl hydrocarbon receptor in environmentally induced skin aging and skin carcinogenesis. Int J Mol Sci. (2019) 20:6005. doi: 10.3390/ijms20236005, PMID: 31795255 PMC6928879

[8] Sklar LR Almutawa F Lim HW Hamzavi IH Uetsu N Miyauchi-Hashimoto H. Shining light on skin pigmentation: the darker and the brighter side of effects of UV radiation. J Invest Dermatol. (2014) 140:573–81. doi: 10.1046/j.1365-2133.1999.02754.x

[9] Bernard JJ Gallo RL Krutmann J. Photoimmunology: how ultraviolet radiation affects the immune system. Nat Rev Immunol. (2019) 19:688–701. doi: 10.1038/s41577-019-0185-9, PMID: 31213673

[10] Huang Y Li Y Hu Z Yue X Proetto MT Jones Y. Mimicking melanosomes: Polydopamine nanoparticles as artificial microparasols. ACS Cent Sci. (2017) 3:564–9. doi: 10.1021/acscentsci.6b00230, PMID: 28691067 PMC5492417

[11] Chang NF Chen YS Lin YJ Tai TH Chen AN Huang CH. Study of hydroquinone mediated cytotoxicity and hypopigmentation effects from UVB-irradiated arbutin and deoxyarbutin. Int J Mol Sci. (2017) 18:969. doi: 10.3390/ijms18050969, PMID: 28467382 PMC5454882

[12] Ahmad I Simanyi E Guroji P Tamimi IA Delarosa HJ Nagar A. Toll-like receptor-4 deficiency enhances repair of UVR-induced cutaneous DNA damage by nucleotide excision repair mechanism. J Invest Dermatol. (2014) 134:1710–7. doi: 10.1038/jid.2013.530, PMID: 24326454 PMC4020975

[13] Moriwaki S Takahashi Y. Photoaging and DNA repair. J Dermatol Sci. (2008) 50:169–76. doi: 10.1016/j.jdermsci.2007.08.011 17920816

[14] Sun X Zhang N Yin C Zhu B Li X. Ultraviolet radiation and Melanomagenesis: from mechanism to immunotherapy. Front Oncol. (2020) 10:951. doi: 10.3389/fonc.2020.00951, PMID: 32714859 PMC7343965

[15] Borkowski AW Kuo IH Bernard JJ Yoshida T Williams MR Hung NJ. Toll-like receptor 3 activation is required for normal skin barrier repair following UV damage. J Invest Dermatol. (2015) 135:569–78. doi: 10.1038/jid.2014.354, PMID: 25118157 PMC4289479

[16] Ding X Willenborg S Bloch W Wickström SA Wagle P Brodesser S. Epidermal mammalian target of rapamycin complex 2 controls lipid synthesis and filaggrin processing in epidermal barrier formation. J Allergy Clin Immunol. (2020) 145:283–300.e8. doi: 10.1016/j.jaci.2019.07.033, PMID: 31401286

[17] He H Li A Li S Tang J Li L Xiong L. Natural components in sunscreens: topical formulations with sun protection factor (SPF). Biomed Pharmacother. (2021) 134:111161. doi: 10.1016/j.biopha.2020.111161, PMID: 33360043

[18] Schuch AP Moreno NC Schuch NJ Menck CFM Garcia CCM. Sunlight damage to cellular DNA: focus on oxidatively generated lesions. Free Radic Biol Med. (2017) 107:110–24. doi: 10.1016/j.freeradbiomed.2017.01.029, PMID: 28109890

[19] Verardo V Gómez-Caravaca AM Arráez-Román D Hettinga K. Recent advances in phospholipids from colostrum, milk and dairy by-products. Int J Mol Sci. (2017) 18:1–23. doi: 10.3390/ijms18010173, PMID: 28106745 PMC5297805

[20] Dai R Hua W Chen W Xiong L Li L. The effect of milk consumption on acne: a meta-analysis of observational studies. J Eur Acad Dermatology Venereol. (2018) 32:2244–53. doi: 10.1111/jdv.15204, PMID: 30079512

[21] Vincenzetti S Polidori P Mariani P Cammertoni N Fantuz F Vita A. Donkey’s milk protein fractions characterization. Food Chem. (2008) 106:640–9. doi: 10.1016/j.foodchem.2007.06.026

[22] Cunsolo V Saletti R Muccilli V Gallina S Di Francesco A Foti S. Proteins and bioactive peptides from donkey milk: the molecular basis for its reduced allergenic properties. Food Res Int. (2017) 99:41–57. doi: 10.1016/j.foodres.2017.07.002, PMID: 28784499

[23] Witkowska-Zimny M Kamińska-El-Hassan E Wróbel E. Milk therapy: unexpected uses for human breast milk. Nutrients. (2019) 11:944. doi: 10.3390/nu11050944, PMID: 31027386 PMC6567207

[24] Zeng L Yang K. Exploring the pharmacological mechanism of Yanghe decoction on HER2-positive breast cancer by a network pharmacology approach. J Ethnopharmacol. (2017) 199:68–85. doi: 10.1016/j.jep.2017.01.045, PMID: 28130113

[25] Qin T Wu L Hua Q Song Z Pan Y Liu T. Prediction of the mechanisms of action of shenkang in chronic kidney disease: A network pharmacology study and experimental validation. J Ethnopharmacol. (2019) 246:112128. doi: 10.1016/j.jep.2019.112128, PMID: 31386888

[26] Shannon P. Cytoscape: A software environment for integrated models of biomolecular interaction networks. Genome Res. (2003) 13:2498–504. doi: 10.1101/gr.1239303, PMID: 14597658 PMC403769

[27] Young AR Narbutt J Harrison GI Lawrence KP Bell M O’Connor C. Optimal sunscreen use, during a sun holiday with a very high ultraviolet index, allows vitamin D synthesis without sunburn. Br J Dermatol. (2019) 181:1052–62. doi: 10.1111/bjd.17888, PMID: 31069787 PMC6899952

[28] Shih BB Farrar MD Cooke MS Osman J Langton AK Kift R. Fractional sunburn threshold UVR doses generate equivalent vitamin D and DNA damage in skin types I–VI but with epidermal DNA damage gradient correlated to skin darkness. J Invest Dermatol. (2018) 138:2244–52. doi: 10.1016/j.jid.2018.04.015, PMID: 29730334 PMC6158343

[29] Gaddameedhi S Selby CP Kemp MG Ye R Sancar A. The circadian clock controls sunburn apoptosis and erythema in mouse skin. J Invest Dermatol. (2015) 135:1119–27. doi: 10.1038/jid.2014.508, PMID: 25431853 PMC4366313

[30] Moore C Cevikbas F Pasolli HA Chen Y Kong W Kempkes C. UVB radiation generates sunburn pain and affects skin by activating epidermal TRPV4 ion channels and triggering endothelin-1 signaling. Proc Natl Acad Sci. (2013) 110:E3225–34. doi: 10.1073/pnas.1312933110, PMID: 23929777 PMC3752269

[31] Biniek K Levi K Dauskardt RH. Solar UV radiation reduces the barrier function of human skin. Proc Natl Acad Sci. (2012) 109:17111–6. doi: 10.1073/pnas.1206851109, PMID: 23027968 PMC3479513

[32] Wang C Wang D Dai T Xu P Wu P Zou Y. Skin pigmentation-inspired polydopamine sunscreens. Adv Funct Mater. (2018) 28:1802127. doi: 10.1002/adfm.201802127

[33] Chan TK Bramono D Bourokba N Krishna V Wang ST Neo BH. Polycyclic aromatic hydrocarbons regulate the pigmentation pathway and induce DNA damage responses in keratinocytes, a process driven by systemic immunity. J Dermatol Sci. (2021) 104:83–94. doi: 10.1016/j.jdermsci.2021.09.003, PMID: 34690024

[34] Lee J Jung E Lee J Huh S Boo YC Hyun CG. Mechanisms of melanogenesis inhibition by 2,5-dimethyl-4-hydroxy-3(2H)—furanone. Br J Dermatol. (2007) 157:242–8. doi: 10.1111/j.1365-2133.2007.07934.x, PMID: 17650175

[35] Serre C Busuttil V Botto JM. Intrinsic and extrinsic regulation of human skin melanogenesis and pigmentation. Int J Cosmet Sci. (2018) 40:328–47. doi: 10.1111/ics.12466, PMID: 29752874

[36] Kocic H Stankovic M Tirant M Lotti T Arsic I. Favorable effect of creams with skimmed donkey milk encapsulated in nanoliposomes on skin physiology. Dermatol Ther. (2020) 33:e13511. doi: 10.1111/dth.13511, PMID: 32372458

[37] Brumini D Criscione A Bordonaro S Vegarud GE Marletta D. Whey proteins and their antimicrobial properties in donkey milk: a brief review. Dairy Sci Technol. (2016) 96:1–14. doi: 10.1007/s13594-015-0246-1

[38] Cha JY Yang HJ Moon HI Cho YS. Branched-chain amino acids complex inhibits melanogenesis in B16F0 melanoma cells. Immunopharmacol Immunotoxicol. (2012) 34:256–64. doi: 10.3109/08923973.2011.600764, PMID: 21854182

[39] Lin YS Wu WC Lin SY Hou WC. Glycine hydroxamate inhibits tyrosinase activity and melanin contents through downregulating cAMP/PKA signaling pathways. Amino Acids. (2015) 47:617–25. doi: 10.1007/s00726-014-1895-8, PMID: 25501504

[40] Chung BY Choi SR Moon IJ Park CW Kim YH Chang SE. The glutathione derivative, GSH Monoethyl Ester, may effectively whiten skin but GSH does not. Int J Mol Sci. (2016) 17:1–11. doi: 10.3390/ijms17050629, PMID: 27128906 PMC4881455

[41] Niu C Yin L Aisa H. Novel furocoumarin derivatives stimulate melanogenesis in B16 melanoma cells by up-regulation of MITF and TYR family via Akt/GSK3β/β-catenin signaling pathways. Int J Mol Sci. (2018) 19:746. doi: 10.3390/ijms19030746, PMID: 29509689 PMC5877607

[42] Ando H Wen ZM Kim HY Valencia JC Costin GE Watabe H. Intracellular composition of fatty acid affects the processing and function of tyrosinase through the ubiquitin-proteasome pathway. Biochem J. (2006) 394:43–50. doi: 10.1042/BJ20051419, PMID: 16232122 PMC1386001

[43] Yamada H Hakozaki M Uemura A Yamashita T. Effect of fatty acids on melanogenesis and tumor cell growth in melanoma cells. J Lipid Res. (2019) 60:1491–502. doi: 10.1194/jlr.M090712, PMID: 31345992 PMC6718436

[44] Okajima S Hamamoto A Asano M Isogawa K Ito H Kato S. Azepine derivative T4FAT, a new copper chelator, inhibits tyrosinase. Biochem Biophys Res Commun. (2019) 509:209–15. doi: 10.1016/j.bbrc.2018.12.105, PMID: 30579605

[45] Grabacka M Plonka PM Urbanska K Reiss K. Peroxisome proliferator—activated receptor α activation decreases metastatic potential of melanoma cells *in vitro* via down-regulation of Akt. Clin Cancer Res. (2006) 12:3028–36. doi: 10.1158/1078-0432.CCR-05-2556, PMID: 16707598

[46] Miklas JW Clark E Levy S Detraux D Leonard A Beussman K. TFPa/HADHA is required for fatty acid beta-oxidation and cardiolipin re-modeling in human cardiomyocytes. Nat Commun. (2019) 10:4671. doi: 10.1038/s41467-019-12482-1, PMID: 31604922 PMC6789043

[47] Blunder S Rühl R Moosbrugger-Martinz V Krimmel C Geisler A Zhu H. Alterations in epidermal eicosanoid metabolism contribute to inflammation and impaired late differentiation in FLG-mutated atopic dermatitis. J Invest Dermatol. (2017) 137:706–15. doi: 10.1016/j.jid.2016.09.034, PMID: 27793761 PMC5551680

[48] Brown SJ McLean WHI. One remarkable molecule: Filaggrin. J Invest Dermatol. (2012) 132:751–62. doi: 10.1038/jid.2011.393, PMID: 22158554 PMC3378480

[49] Thyssen JP Kezic S. Causes of epidermal filaggrin reduction and their role in the pathogenesis of atopic dermatitis. J Allergy Clin Immunol. (2014) 134:792–9. doi: 10.1016/j.jaci.2014.06.014, PMID: 25065719

