# Supplementary material for: Effects of donkey milk on UVB-induced skin barrier damage and melanin pigmentation: A network pharmacology and experimental validation study
Source: Front Nutr. 2023 Mar 9;10:1121498. doi: 10.3389/fnut.2023.1121498 (PMC10033878; doi:10.3389/fnut.2023.1121498)
Supplement: Supplementary file 1 [file Data_Sheet_1.DOCX]

**Effects of donkey milk on UVB‑induced skin barrier damage and melanin pigmentation: a network pharmacology and experimental validation study**

Anqi Li ^1,2,3^, Hailun He ^1,2,3^, Yanjing Chen^1,2,^^3^, Feng Liao^4^, Jie Tang^1,2^, Li Li^5^, Yumei Fan^4^, Li Li ^1,2,3*^, Lidan Xiong^1,2*^

^1^ Cosmetics Safety and Efficacy Evaluation Center, West China Hospital, Sichuan University, Chengdu, Sichuan, China

^2^ NMPA Key Laboratory for Human Evaluation and Big Data of Cosmetics, Chengdu, China

^3^ Department of Dermatology, West China Hospital, Sichuan University, Chengdu, Sichuan, China

^4^ National Engineering Research Center for Gelatin-based Traditional Chinese Medicine, Dong-E-E-Jiao Co. Ltd., Shandong, China

^5^ Laboratory of Pathology, West China Hospital of Sichuan University, Chengdu, Sichuan, China

*Correspondence: xionglidan@wchscu.cn (Lidan Xiong), hxskincos2017@qq.com (Li Li)

**MATERIALS AND METHODS**

**Materials and Reagents**

**Determination of Mineral Content**

The ICP-OES operating conditions are listed in Table S1.

Table S1 ICP-OES operating conditions

| Operating conditions | |
| --- | --- |
| Power | 1500 W |
| Plasma gas flow | 15 L/min |
| Carrier gas flow | 0.80 L/min |
| Auxiliary air flow | 0.40 L/min |
| Helium flow | 4 mL/min |
| Sample aspiration rate | 0.3 r/s |
| Spray chamber temperature | 2 ℃ |

**Determination of vitamin C, vitamin D2 and vitamin D3 content**

The concentration of vitamin C was measured by the method provided by the National Standards of the People’s Republic of China (PRC). Briefly, to determine the ascorbic acid content, DM was mixed with metaphosphoric acid in alcohol to precipitate the proteins and stabilize the ascorbic acid. Analysis of vitamin C was performed by fluorospectrophotometry and the fluorescence was measured with immission at 270 nm and emission at 315 nm.

For the determination of vitamin D, DM was saponified by adding KOH (50% w/w) according to the method described by the National Standards of the PRC. Saponification occurred at 80°C for 30 min. Afterward, the solution was transferred to a 250 mL separatory funnel, and an initial extraction of the unsaponifiable fraction was performed using 50 mL of ligarine. The aqueous phase was thus drained and collected to repeat for twice extractions by adding 50 mL of ligarine each time. Each time, the organic phase was collected in a rotary evaporator flask and finally evaporated to dryness on a rotary evaporator. The extract was resuspended in 2 mL of hexane and filtered through a 0.22 μm diameter syringe filter. 100 μL of the extract was injected into a high performance liquid chromatographic (HPLC) instrument and isocratically eluted using as a mobile phase methanol-water 95:5 at a flow of 1 mL/min. A Kinetex core-shell column (Phenomenex Inc., Torrance, CA) was used as the stationary phase and the UV detector was set at 264 nm. Vitamins D2 and D3 in the DM samples were quantified by comparison with a calibration curve obtained with the injection of the pure standards (Sigma-Aldrich, St. Louis).

**Determination of** **taurine content**

Taurine in DM was determined by HPLC analysis. HPLC analysis were applied by a Thermo U3000 (Thermo Fisher Scientific, Pittsburgh, PA, USA) system with a UV detector set at 254 nm. The chromatographic column (Intertsil ODS-SP C18, Japan) was performed. After sample pretreatment, a gradient mobile phase system consisting of sodium acetate buffer (phase A), chromatogram acetonitrile (phase B) at a ratio of 70:30, a flow rate of 1.0 mL/min and 20 μL of DM for 15 min at 25℃ was injected.
**Determination of** **phosphorus content**

Samples were analyzed for phosphorus concentration using phosphovanoclonolybeate molecular absorption spectrometric method. Samples were placed in a Gallenkamp muffle furnace for 4 h at 550°C, then added with 2 mL of 50% (v/v) HCl and diluted with deionized water to 100 mL. After 10 mL of diluent and an equal amount of blank solution were transferred to a 50 mL volumetric flask, 6 mol/L of sodium hydroxide solution, 0.2 mol/L nitric acid solution, 0.1 mol/L of sodium hydroxide solution and 10 mL of ammonite vanadate molybdate intermixture were added in turn. The solution was diluted to a total of 50 mL with water. The concentrations of phosphorus in DM were analyzed by ultraviolet-visible spectrophotometry (UV-Vis) at 440 nm.

**Determination of** **chloride content**

The determination of chloride in DM were executed by a Zeta potentiometric titrator concerned. Sample pretreatment needed to be carried out before titration. 70 mL of heat-distilled water was added to 10 g of DM power sample, and then the sample solution was heat-treated in a water bath at 100°C for 15 min. After the sample solution cooled to room temperature, 2 mL of 0.25 mol/L potassium ferrocyanide solution and 2 mL of 1.2 mol/L zinc acetate solution were added in turn for a 30 min reaction. Then the solution was diluted to 100 mL with water and filtered. After the pretreatment, we added 10 mL of diluent , 5 mL of 0.25%（v/v）of nitric acid solution and 25 mL of acetone, respectively, in a 50 mL beaker, then the chloride ions in diluent were titrated by using 0.02 mol/L of silver nitrate standard solution.

**Results**

**The outcomes of** **AAs contents in DM powder**

The evolution of total AAs of DM powder is shown in Figure S1. AAs were identified according to their retention times and by comparison with those of the standard mixture solution. The AAs contents of DM powder are shown in Table S2. Of the 16 amino acids identified, the most abundant were glutamic acid (Glu), Aspartic acid (Asp), leucine (Leu), lysine (Lys), valine (Val) and Arginine (Arg), which accounted for over 66% of the total AAs.


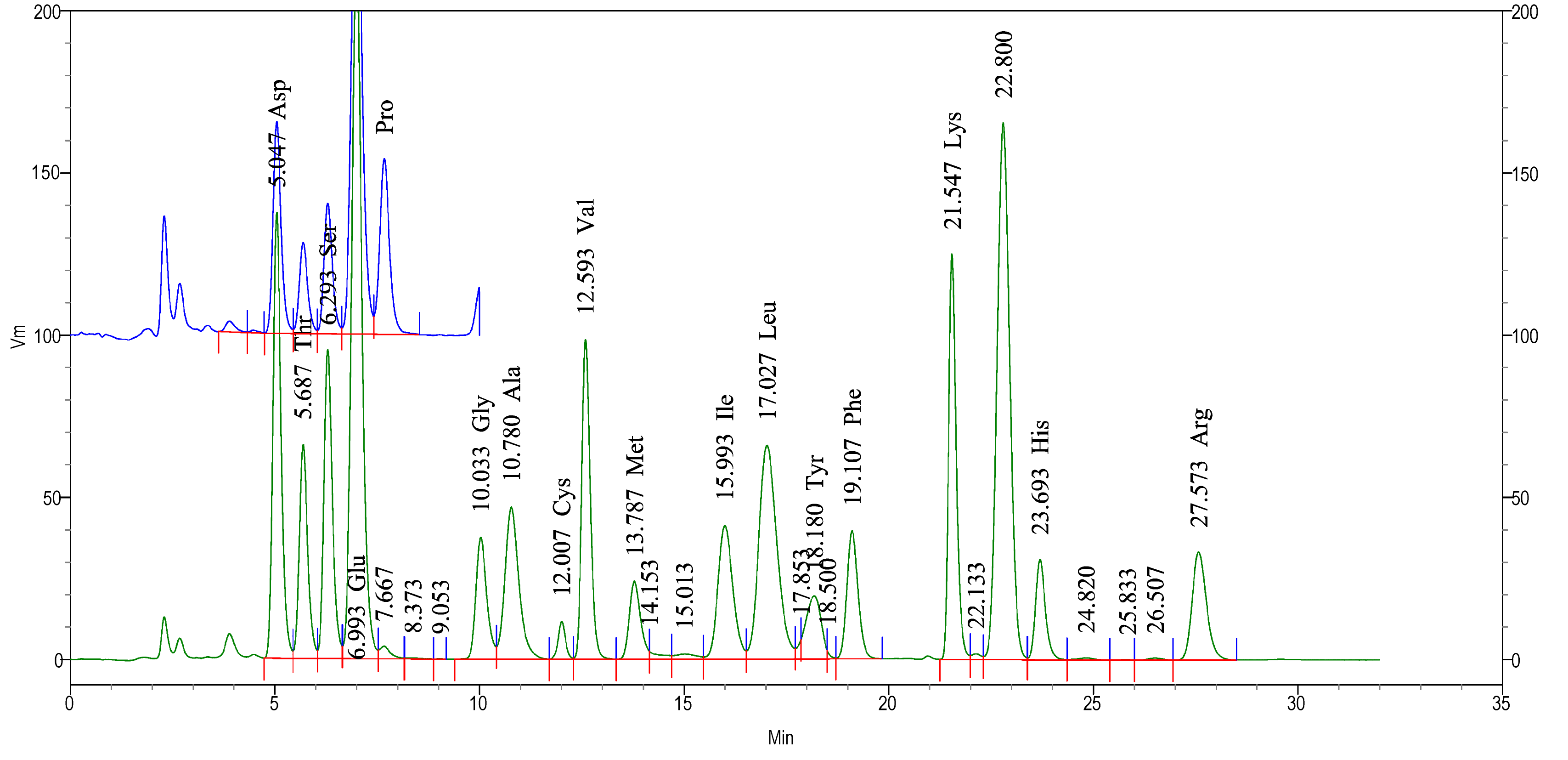


Fig. S1. HPLC chromatogram of amino acids in DM

Table S2 Amino acids of DM

| RT | Item | Value (μg/ml) |
| --- | --- | --- |
| 5.047 | Aspartic acid (Asp) | 33.355 |
| 5.687 | Threonine (Thr) | 13.987 |
| 6.293 | Serine (Ser) | 17.528 |
| 6.993 | Glutamic acid (Glu) | 67.704 |
| 10.033 | Glycine (Gly) | 6.522 |
| 10.780 | Alanine (Ala) | 12.058 |
| 12.007 | Cysteine (Cys) | 3.992 |
| 12.593 | Valine (Val) | 20.458 |
| 13.787 | Methionine (Met) | 8.319 |
| 15.993 | Isoleucine (Ile) | 16.721 |
| 17.027 | Leucine (Leu) | 32.011 |
| 18.180 | Tyrosine (Tyr) | 11.507 |
| 19.107 | Phenylanaline (Phe) | 16.035 |
| 21.547 | Lycine (Lys) | 26.355 |
| 23.693 | Histidine (His) | 9.579 |
| 27.573 | Arginine (Arg) | 18.004 |

**The outcomes of** **fatty acids contents in DM powder**

In order to investigate the fatty acid profile in DM, the content of fatty acids in DM was analyzed by GC. The results showed that 28 kinds of fatty acids were detected in DM (Figure S2). The content of fatty acids of DM powder was 4.5%. The content of saturated fatty acids and unsaturated fatty acids was 2.16% and 2.34% respectively. (1.12% for unsaturated fatty acids and 1.22% for polyunsaturated fatty acid). The content of unsaturated fatty acids of DM, including myristoleic (C14:1), palmitoleic(C16:1), trans-elaidic(C18:1n9t), oleic (C18:1n9c), linoleic (C18:2-9c,12c), α-linolenic acid (C18:3; ALA), cis 11-eicosenoic acid (C20:1n11c), all cis-11,14-eicosadienoic acid (C20:2-11,14c), all cis-8,11,14-eicosatrienoic acid (C20:3-8,11,14c), all cis-5,8,11,14-eicosateraenoic acid (C20:4-5,8,11,14c; ARA), all cis-13,16-docosadienoic acid (C22:2-13,16c), nervonic(C24:1).


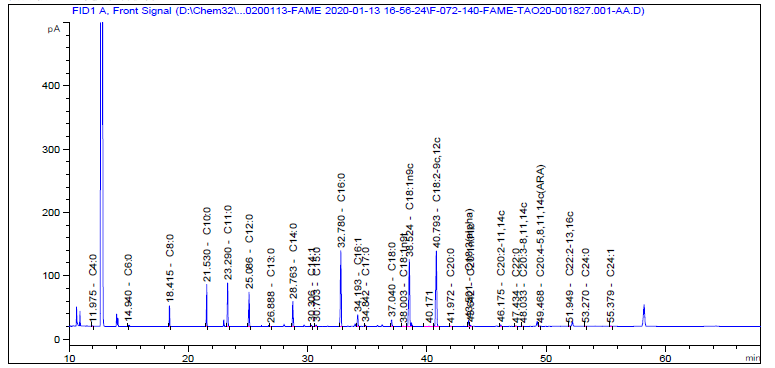


Fig. S2. GC chromatogram of fatty acids in DM

**The outcomes of vitamin C, vitamin D2 and vitamin D3 in DM**

Total vitamin C and vitamins (vitamin D2 and vitamin D3) concentrations found in DM powder were 48.1 mg/100 mg and ND respectively.

**The outcomes of** **mineral in DM**

The contents of six kinds of elements（K, Ca, Na, Mg, Fe, Zn）in DM were determined by ICP-OES. DM was digested with microwave digestion procedure. The results showed that DM had the highest Ca, K and Na content (8.81 g/kg, 8.27 g/kg and 2.59 g/kg, respectively). The content of Mg and Zn in DM were 826 mg/kg and 22.7 mg/kg, respectively. It is worth noting that Fe wasn’t detected in the DM.

**The outcomes of taurine and cholesterol** **in DM** **powder**

Taurine was separated under the mobile phase comprised sodium acetate buffer and chromatogram acetonitrile (70:30) for 15 min at 25℃ with a flow rate of 1 mL/min and measured by UV detector at 254 nm when the absorption peak was at 10.943 min (Figure S3). The content of taurine in DM powder was 12.3 mg/100 g.


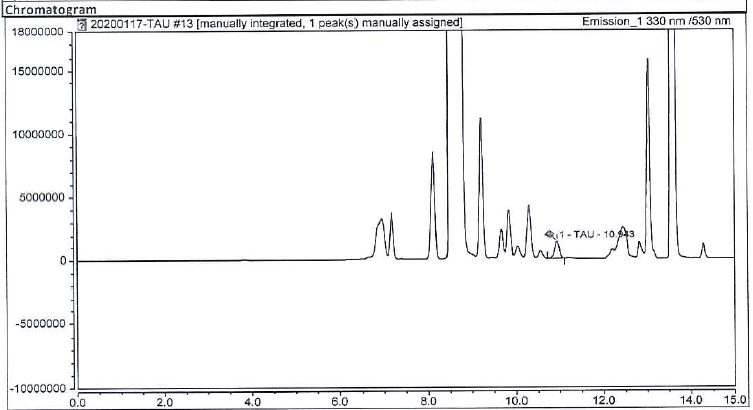


Fig. S3. HPLC chromatogram of taurine in DM

Cholesterol was separated under the condition that the mobile phase methanol for 30 min at 38℃ with a flow rate of 1 mL/min and measured by UV detector at 205 nm when the absorption peak was at 12.653 min (Figure S4). The results revealed that the contents of cholesterol in DM powder was 21.7 mg/100 g.


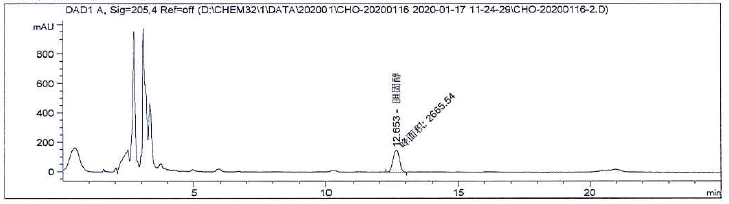


Fig. S4. HPLC chromatogram of cholesterol in DM

**The outcomes of phosphorus content in DM** **powder**

Samples were analyzed for phosphorus concentration using fluorospectrophotometry molecular absorption spectrometric method. The level of P in DM power was 580 mg/100 g.

**Determination of chloride content**

Chloride ion concentration in DM was determined by a potentiometric titrator concerned. The result showed that chloride concentrations found in DM power was 0.58 g/100 g.
